# Elevated Adrenocorticotropic Hormone After Adrenalectomy or Adrenal Ablation and Immune Checkpoint Inhibitors: Adrenal Insufficiency Is Not Always the Culprit

**DOI:** 10.7759/cureus.85573

**Published:** 2025-06-08

**Authors:** David Shabsovich, Nikhita Kathuria-Prakash, Jiajia Zhang, Marsenne Cabral, Lidia Lopez, Amit Sumal, Alexandra Drakaki

**Affiliations:** 1 Internal Medicine, University of California Los Angeles, Los Angeles, USA; 2 Hematology-Oncology, University of California Los Angeles, Los Angeles, USA; 3 Endocrinology, University of California Los Angeles, Los Angeles, USA

**Keywords:** acth, adrenalectomy, adrenal insufficiency (ai), cancer immunotherapy, hematology-oncology

## Abstract

Adrenal insufficiency is a known complication of both immune checkpoint inhibitors (ICIs) and resection or ablation of the adrenal glands. In the modern era of immunotherapy, more frequent assessment of the adrenal hormonal axis is performed after initiation of ICIs in order to monitor for such complications. The interpretation of these laboratory tests, such as elevated adrenocorticotropic hormone (ACTH), in patients who receive ICIs and undergo adrenalectomy or adrenal ablation provides an additional set of diagnostic challenges that are not well described. We present a case series of four patients who had elevated ACTH without clinical (symptoms) or biochemical (decreased cortisol) evidence of adrenal insufficiency, a pattern suggesting a compensatory increase in ACTH. This highlights an emerging phenomenon and diagnostic challenge in the monitoring of the adrenal axis in patients undergoing adrenalectomy or ablation alongside ICI therapy, emphasizing the need for close follow-up and thorough investigation to rule out adrenal insufficiency in such patients.

## Introduction

Although endocrinopathies after therapy with immune checkpoint inhibitors (ICIs) for treatment of various malignancies are common and well-established by means of immune-mediated destruction, primary adrenal insufficiency is relatively rare, accounting for less than 2% of ICI-related endocrinopathies [1]. Treatment with adrenal resection or ablation is also independently associated with adrenal insufficiency, although in the absence of pre-operative excess of cortisol production, this is likewise less likely [2]. In the modern era of cancer therapy, patients often receive multiple treatment modalities, including systemic therapies in combination with local interventions such as metastasectomies, radiation, and ablative procedures. Therefore, when an ICI is offered in subjects who have undergone adrenal resection or ablation, the interpretation of adrenal axis hormonal profiles becomes challenging. In particular, the elevation of plasma adrenocorticotropic hormone (ACTH) is an emerging phenomenon that can lead to diagnostic uncertainty. Although this finding may suggest a disorder of the adrenal axis, it may also represent a compensatory increase in ACTH without evidence of adrenal insufficiency clinically and on assessment of morning serum cortisol levels, as observed in our case series.

## Case presentation

We surveyed a database of all patients who underwent ICI therapy for any indication at our institution from January 1, 2015 to March 1, 2024. Using the International Statistical Classification of Diseases and Related Health Problems, 10th revision (ICD-10) codes, we identified patients who underwent either adrenalectomy or adrenal ablation. Among those, we excluded patients who had normal ACTH, had a known history of an adrenal axis disorder, or were on chronic steroid therapy. Upon identification of the study cohort, we collected and analyzed data about their malignancies, treatment courses, and adrenal axis hormone profiles. This study, which involved human subjects, was approved by the UCLA Institutional Review Board.

Four patients were identified who received ICI therapy in addition to adrenal resection or ablation and had elevated ACTH without clinical or biochemical evidence of primary adrenal insufficiency.

Patient 1

A 74-year-old man with a history of metastatic clear cell renal cell carcinoma underwent left nephrectomy and adrenalectomy followed by treatment with a programmed death-ligand 1 (PD-L1) inhibitor, which was started four months after his surgery. He developed an osseous metastasis to the right radius which was surgically resected and then a vascular endothelial growth factor (VEGF) inhibitor was added to his treatment regimen. He was noted to have elevated ACTH levels (103 pg/dL, normal range 6 to 59 pg/dL) with normal morning serum cortisol (18 mcg/dL, normal range 8 to 25 mcg/dL). Elevated ACTH was initially noted 106 months after surgery, while still receiving combination therapy.

Patient 2

A 58-year-old man with a history of metastatic Merkel cell carcinoma underwent resection of the primary tumor followed by adjuvant radiation therapy, had interatrial recurrence for which he underwent an additional resection, and then had cerebellar metastases for which he underwent further radiation therapy. He then received treatment with a programmed cell death protein 1 (PD-1) inhibitor which was stopped due to ICI-related hepatitis. The patient then developed left adrenal metastasis for which he underwent left adrenalectomy, eight months after initiation of ICI therapy. He was noted to have elevated ACTH (117 pg/dL) and normal serum cortisol (13 mcg/dL, assessed in the afternoon) five months after initiation of ICI therapy and three months before his adrenal intervention. Two months after the adrenal intervention, ACTH was 127 pg/dL and cortisol was 8 mcg/dL (also assessed in the afternoon).

Patient 3

A 61-year-old woman with a history of oligometastatic clear cell renal carcinoma presented with left renal and right adrenal masses. The adrenal mass was thought to be an adenoma initially based on computed tomography imaging findings so the patient underwent left nephrectomy. Eventually, she underwent biopsy of the right adrenal mass which was consistent with metastatic clear cell renal carcinoma. The patient was started on a first-line PD-1 inhibitor in combination with a VEGF inhibitor followed by right adrenalectomy 2 months later. Six months after treatment initiation, she was noted to have elevated ACTH (73 pg/dL) with morning serum cortisol of 19 mcg/dL.

Patient 4

A 77-year-old man with a history of early-stage hepatocellular carcinoma underwent surgical resection at the time of diagnosis. Subsequently, he developed local recurrence and metastatic disease in the left adrenal gland. He underwent additional liver resection as well as ablation of the left adrenal gland. Eventually, he developed pulmonary metastases for which he was initially started on a VEGF inhibitor. At the time of progression, he was switched to a combination of a PD-L1 inhibitor and a VEGF inhibitor. His course was further complicated by several intrahepatic recurrences treated with ablations and right adrenal metastasis that was managed with radiation therapy. ACTH was noted to be elevated (108 pg/dL) 74 months after his initial adrenal intervention with morning serum cortisol at that time being normal at 17 mcg/dL.

Patient characteristics are further summarized in Table 1. Follow-up for patients ranged from 3 to 23 months.

**Table 1:** Characteristics and temporality of ACTH elevation in patients who received ICI therapy and adrenal resection or ablation without clinical or biochemical evidence of adrenal insufficiency ACTH: Adrenocorticotropic hormone; ICI: immune checkpoint inhibitor

Patient	Age/Sex	Primary Malignancy	Adrenal Intervention	Systemic Cancer Treatments	Time between Adrenal Intervention and Elevated ACTH (Months)	Time between ICI Initiation and Elevated ACTH (Months)
1	74/M	Renal cell carcinoma (clear cell)	Left adrenalectomy	Atezolizumab, bevacizumab	106	110
2	58/M	Merkel cell carcinoma	Left adrenalectomy	Pembrolizumab	2	10
3	61/F	Renal cell carcinoma (clear cell)	Right adrenalectomy	Lenvatinib, pembrolizumab	4	6
4	77/M	Hepatocellular carcinoma	Left adrenal ablation, right adrenal radiation therapy	Sorafenib, atezolizumab, bevacizumab, lenvatinib	74	20

## Discussion

Here, we present a case series of patients who underwent both ICI therapy and adrenal interventions (resection or ablation) and were found to have elevated ACTH of unclear clinical significance, but without clear clinical or biochemical evidence of adrenal insufficiency. Causes of elevated ACTH include primary adrenal insufficiency, Cushing syndrome from an ACTH-producing pituitary adenoma or ectopic ACTH production, congenital adrenal hyperplasia, or assay interference. More recently, the phenomenon of compensatory ACTH elevation in the absence of adrenal insufficiency has been described in patients undergoing unilateral adrenalectomy. Honda et al. described a series of patients undergoing unilateral adrenalectomy for primary hyperaldosteronism, and although ACTH levels were significantly increased from baseline post-operatively, no patient in their series developed frank adrenal insufficiency [3]. Yokohama et al. also evaluated the adrenal axis in patients with renal cell carcinoma with adrenal involvement who underwent adrenalectomy and likewise found a significant elevation in ACTH post-operatively; they did not find any differences in cortisol levels post-operatively, but did note differences in cortisol kinetics after ACTH stimulation between patients who underwent adrenal intervention and those who did not [4]. Finally, Shen et al. reported on the need for steroid therapy in a series of 331 patients undergoing adrenalectomy and found that no patient undergoing adrenalectomy for non-Cushing adrenal disease developed clinical adrenal insufficiency or needed steroid therapy [5].

The incidence of ICI-related primary adrenal insufficiency is relatively low. In a meta-analysis assessing 38 randomized clinical trials comprising over 7,500 patients, Barroso-Sousa et al. identified a total of 43 cases of any grade [6]. Shi et al. performed a systematic review and identified 15 published cases of ICI-related primary adrenal insufficiency, highlighting its relative rarity, association with other ICI-related endocrinopathies, and high mortality and morbidity if not promptly recognized [7]. Given the low incidence of ICI-related primary adrenal insufficiency, an elevated ACTH in patients who undergo ICI therapy in conjunction with other adrenal interventions should be followed up with at least serum cortisol testing to assess for adrenal insufficiency. However, given the possibility of post-operative compensatory ACTH elevation in patients who undergo adrenal interventions, a substantial proportion of patients undergoing ICI and adrenal interventions may in fact have a compensatory ACTH elevation that can be monitored without clinical intervention. Interestingly, Bando et al. assessed ACTH levels in a cohort of patients receiving ICI and found that fluctuations in ACTH levels after ICI may be associated with increased risk of ICI-related hypophysitis [8]. Therefore, the clinical consequences and implications of these findings, particularly in patients who have received both ICI and an adrenal intervention, are not well described or understood to date.

## Conclusions

Elevated serum ACTH after immunotherapy and adrenal intervention is an emerging phenomenon of unclear diagnostic and prognostic significance that is more prevalent in the modern era of cancer treatment. This series and review of the literature highlight that in a substantial number of cases, such elevations in the absence of clinical or biochemical evidence of adrenal insufficiency may represent a compensatory increase in ACTH that requires monitoring without clinical intervention. Further data is needed to better characterize ACTH elevation in this setting, understand its clinical consequences, and appropriately monitor and risk stratify patients.
